# Refutation of Certain Charges in Sir Wm. Adam's Late Publication

**Published:** 1818-03

**Authors:** John Stevenson


THE LONDON
### Medical and Physical Journal.

3 OF VOL. XXXIX.] MARCH, 1818. [NO. 229.

"For many fortunate discoveries in medicine, and for the detection of numerous errors, the world is indebted to the rapid circulation of Monthly "Journals; and there never existed any work to which the Faculty in "EUROPE and AMERICA were under deeper obligations than to the "Medical and Physical Journal of London, now forming a long, but an "invaluable, series."—RUSH.

*For the London Medical and Physical Journal.*

### *Refutation of certain Charges in Sir Wm. Adam's late Publication.* By JOHN STEVENSON, Esq.

I CANNOT but consider it just cause for self-congratulation, that Sir William Adams, whose name I have never introduced into any of my publications, and with whom I have not the slightest personal acquaintance; but who seems, from the list of highly respectable medical practitioners he has made the objects of his animadversions, to be a man

"Jealous in honours, sudden and quick in quarrel;"

after scrutinising my conduct and successful career with eagle-eye, is at length able only to bring against me the truly grievous charge of having, above seven years since, anticipated him in the alteration and use of a surgical instrument! The accusation is, indeed, so frivolous and unimportant, that, had I not been called upon in the Review of Sir William's recent publication, which appeared in the last number of your Journal, in which his pretensions to originality are duly appreciated; and deemed it possible that my silence might be disadvantageously interpreted by some of the numerous extra-professional persons, amongst whom the Letter and Supplement, containing "the ground and front of my offending," have been gratuitously distributed by the author, I should not have sacrificed that time in noticing it, which can be so much more agreeably and beneficially employed. But this allegation even, insignificant as it is in itself, when stripped of the imposing effect of dates, garbled quotations, and gratuitous assertions, is founded upon a presumption as improbable and extravagant as, from the sequel, it will be found untrue. For, can it be conceived for a moment, by the most credulous, that an apothecary, whatever his talents and inclination, after once only witnessing an operation for cataract, which he had never before seen, could be capable of impressing my mind with such a distinct idea of the several steps of that process, and of the particular form and mechanism of the instruments, &c. &c. as to enable me accurately to detail the one, and to describe and delineate the other?"

Conclusive as this view of the question may seem, my exculpation does not rest on merely presumptive evidence, or speculative and abstract reasoning. I have had an interview with the apothecary (whose address I fortunately procured through the medium of a patient, to whom the aforesaid pamphlets were sent, with the name, which is carefully suppressed in the text, written by the pen of Sir William himself at the foot of the page), who is made, without his knowledge, to act so prominent a part in this singular drama, and upon whose presumed communication the whole weight of Sir William's argument, and my implied guilt, principally depend. That worthy and highly respectable gentleman "authorises me to state, and to refer any one to him for further information, that, though as a casual and disinterested visitor on the occasion alluded to, he had no motive for retaining in his recollection the particulars of a transaction which took place so many years ago, and with which he had not the smallest concern, he has no hesitation in declaring his conviction, as he could not from what he might observe, or from his own previous knowledge of the operation, hope to add any thing to my intimate acquaintance with the subject, that the insinuation, as far as concerns me, is not only purely imaginary, but utterly destitute of foundation!" This declaration, which I can conscientiously confirm, at once acquits me of having obtained, through that channel, the information relative to the construction and mode of using my needle, so illiberally imputed to me by Sir William Adams!

I might also add, as a further proof, if any were wanted, of the groundlessness of the aforesaid charge, that "the curious coincidence," on which Sir William lays so great stress, between the period when he performed the operation in Portman-square,—viz. in March 1811,—and the time at which the alteration in Mr. Saunders' needle is stated to have been directed by me, applies by virtue only of the *omission*, on the part of Sir William, of *two small*, but on this present occasion not unimportant *words*, which are inserted p. 77 of the first edition of my "Practical Treatise on Cataract," from which the passage in question is derived. As these unlucky and discarded words,—namely, "upwards of,"—were intended to convey an indefinite meaning, not imagining at the time they were written that extreme accuracy on the score of dates was indispensable, the following note from my instrument-maker, Mr. Ewing,

(who has prepared, and will be ready to exhibit, comparative specimens of Sir William's and my needle and speculum, and also various surgical instruments altered and invented by me, for the purpose of facilitating different operations on the eye and ear,) will show, not only that my needle was in being, and of its present shape too, many months prior to the earliest period assigned for its construction by Sir William upon the most vague surmises, but that it differs likewise in one important point of its mechanism from that of Sir William Adams. The cases which suggested that improvement, and which occurred so long since as the year 1809, with the advantages resulting from the subsequent alteration of the needle, are explained at large (with several particulars relating to the nature of the disease, to black cataract, and the after-treatment, &c. not even adverted to by Sir William,) at page 103 and 147 of the second edition of my "Practical Treatise on Cataract."*

"*No.* 1, *Drury-lane*; *Feb.* 14, 1818.

"Sir,

"I am ready to assert, that your needle was made of its *present form* in the year 1810, and that it may be distinguished by being *round* at the part where Sir William Adams' is *flattened.*

"I am, Sir, your very obedient servant,

Wm. Ewing."

"*To John Stevenson, Esq.*"

With regard to my *Speculum*, which has been always represented as an *alteration only* of Pellier's, little need be said, since I have long discontinued the use of any kind of *mechanical* assistance for steadying the Eye. I cannot however forbear observing that, had Sir William taken the trouble only to insert my description of it, as given at page 129 of the second edition of my "Treatise," and the faithful delineations prefixed to that work, instead of presenting us, as he has done, with a *simple wooden outline* sketch, which conveys just as exact a notion of its real form and character, as a circle would of the figures and superscription on the face of a medal, I should have escaped all suspicion of plagiarism.

---

* This work, which is published by Longman and Co. Paternoster-row, and Harwood, Great Russel-street, contains likewise a well-engraved plate of my speculum, my iris or capsule knife, and of my needle, as well as a copy of that of Mr. Saunders', with the *only authentic document*, relative to his modes of operating for the different species of cataract, which is left on record, and was communicated to me in a letter by my friend, that late ingenious surgeon.

What has been advanced is, I presume, quite sufficient to refute the unwarrantable charges brought against me by Sir William Adams, of having availed myself, "not only of his operation for the soft cataract, but likewise of the instruments employed by him in its execution."

In short, had he not, in his equally unprovoked and unjustifiable attack upon me, wantonly violated the common principles of justice, I should have been spared the irksome task of offering this public appeal in vindication of those claims, which are proved to have existed long before any publication of Sir William Adams had issued from the press; the earliest of which (on Diseases of the Eye) did not appear until the middle of August, 1812!

"Sed magna est veritas, et prævalebit."

*Great Russel-street;*
*Feb.* 16, 1818.

---

*For the London Medical and Physical Journal.*

